# Challenges, barriers and opportunities for neglected tropical disease programs of conflict-affected African countries

**DOI:** 10.1093/inthealth/ihaf149

**Published:** 2026-05-08

**Authors:** Emma M Harding-Esch, Francesca Bird, Ambreen Bashir, Louise A Kelly-Hope

**Affiliations:** Clinical Research Department, London School Hygiene and Tropical Medicine, Keppel Street, London WC1E 7HT, UK; Clinical Research Department, London School Hygiene and Tropical Medicine, Keppel Street, London WC1E 7HT, UK; Clinical Research Department, London School Hygiene and Tropical Medicine, Keppel Street, London WC1E 7HT, UK; Department of Livestock and One Health, Institute of Infection, Veterinary and Ecological Sciences, University of Liverpool, Brownlow Hill, Liverpool L3 5RF, UK

**Keywords:** conflict, neglected tropical diseases, sub-Saharan Africa, violence

## Abstract

**Background:**

Insecurity and conflict threaten achievement of global neglected tropical disease (NTD) targets. Identifying conflict-associated challenges and mitigation strategies can inform NTD programmatic decision-making.

**Methods:**

In-depth interviews were conducted with key stakeholders to explore their experiences of implementing NTD programs in conflict-affected regions in sub-Saharan Africa. Study 1 included a variety of stakeholders in Nigeria and study 2 included the NTD program managers from multiple countries. Thematic analysis was used to identify and extract key themes.

**Results:**

Fourteen participants (seven participants per study) from six countries and several NTD programs reported an array of complex challenges that were categorised into three common program-specific activity themes (implementation, community engagement, sustainability) and five common external influencing themes (access to areas; water, sanitation and hygiene; inclusivity; stakeholder partnering; financial resources). Study 1 included a unique theme for solutions. Several common subthemes (surveillance, community education, displaced populations, access for women) and unique study 1 subthemes were reported.

**Conclusions:**

Many complex interacting factors hinder NTD programs in conflict-affected NTD-endemic areas. The scale and breadth of the common and unique challenges and potential solutions require more investigation to enable the development of a practical framework to support programs in achieving the global NTD goals of 2030.

## Introduction

Neglected tropical diseases (NTDs) are a group of 21 diseases and conditions that are an ongoing source of debilitating health and social and economic burden to some of the most marginalised and vulnerable communities worldwide. The prevalence, distribution and disease burden of NTDs are high in sub-Saharan Africa, accounting for more than one-third of the global burden.^1^ In the last decade, NTDs have become a global health priority and the NTD Road Map 2021–2030, published by the World Health Organization (WHO), defines the global targets to control, eliminate or eradicate all NTDs, which are aligned with the sustainable development goals.^2,3^

WHO has classified two broader groups for the majority of NTDs, based on the recommended method of management.^1,2^ Case-management NTDs (Buruli ulcer, human African trypanosomiasis, rabies, guinea worm disease, lymphoedema, leprosy, trichiasis, leishmaniasis, yaws, dengue and mycetoma) are diseases that require individual-level care due to their management complexity or lack of effective diagnostic and treatment tools. Preventative chemotherapy NTDs (onchocerciasis, schistosomiasis, lymphatic filariasis, trachoma and soil-transmitted helminths) can be treated through mass drug administration (MDA) to entire populations, aiming to reduce morbidity and prevent further transmission of these diseases. Preventative chemotherapy is also implemented alongside vector control; water, sanitation and hygiene (WASH) services; and veterinary public health services to support and improve treatment outcomes. The NTDs that do not fall into these two categories, such as rabies, snakebite and dengue, have different approaches to prevention and control.

The long-term nature of tackling many NTD infections and diseases is further compromised by limited financial and human resources and access to healthcare services in many low-income countries, especially in Africa.^4^ In addition, NTD programs are hampered by conflict and political instability that disrupt societal structures and healthcare services, resulting in strains on the health infrastructure, including damaged buildings and disruption to essential facilities such as WASH; unintended mass population movement, including refugees and internally displaced persons living in unsanitary and crowded conditions and vulnerable to opportunistic infections; and a diversion of essential funding.^5–9^ These challenging factors can alter the epidemiology of NTDs and negatively impact the effectiveness of program activities, including conducting prevalence surveys and other surveillance activities and implementing interventions such as preventive chemotherapy and patient clinical care, as well as WASH-related activities.^10–12^

Currently there are no formal recommendations for NTD programs operating in conflict settings, however, there is increasing recognition among stakeholders that it is a critical challenge that needs addressing to ensure that ‘no one is left behind’ and the NTD Road Map goals are achieved and aligned with the United Nations Sustainable Development Goals.^2,11–16^ There has been little research to examine how conflict events such as civil war, terrorism or military coups directly affect NTD programs and how programs have responded to these challenges. Therefore, this study aimed to engage with individuals involved with NTD program management and implementation activities as an important first step to determine the range of common and context-specific challenges and potential mitigating strategies.

## Methods

### Study overview

Two separate, but complementary, qualitative studies in conflict-affected countries in Africa were conducted in July–August 2023. Study 1 focused on one country, Nigeria, to gain an in-depth exploration within a single setting. A variety of key stakeholders, defined as individuals working for organisations supporting NTD programs in Nigeria, participated in the study. Study 2 focused on multiple countries and involved one NTD program manager in each country. This enabled cross-country diversity and commonality of perspectives and experiences to be identified.

### Participant recruitment

A proposed sample size of 10–15 participants was targeted for each study, or when saturation was reached. For study 1, key stakeholders working on NTD programs in Nigeria were recruited. Initial participants were identified through already established professional contacts, with subsequent stakeholders identified using snowball sampling from the initial participants through networks within and between organisations working on NTD programs in Nigeria. For study 2, the NTD program managers from 16 African countries that were classified as high-conflict and/or high-NTD-burden countries were invited to participate.^11,12^ These participants were identified through professional networks, including through the NTD Non-governmental organisation (NGO) Network (NNN) Conflict and Humanitarian Emergencies (C&HE) working group.^17^ Participants were identified through purposive sampling based on their professional roles^18^ and snowball sampling.^19^ The countries invited to participate in study 2 included Angola, Burkina Faso, Burundi, Cameroon, Central African Republic (CAR), Chad, Democratic Republic of the Congo (DRC), Ethiopia, Mali, Mozambique, Niger, Nigeria, Kenya, South Sudan, Tanzania and Uganda.

### Data collection

Participants were invited by e-mail to participate in semi-structured interviews, using predetermined interview topic guides (Supplementary Information 1). This design allowed participants to reveal information that may have not been considered by the researcher prior to the interview.^20^ The topic guides were developed from an initial review of the literature on NTD programs in conflict-affected countries, with a set of themes and open-ended questions established to guide the interview. The topic guide was further refined through feedback from the NNN C&HE working group^17^ and the International Coalition for Trachoma Control Special Populations task team.^16^

Interviews were conducted and recorded using online video conferencing (for study 1, Microsoft Teams computer software [Microsoft, Redmond, WA, USA; https://www.microsoft.com/en-gb/microsoft-teams/group-chat-software] and for study 2, Zoom [Zoom Video Communications; https://zoom.us]) and lasted 30–60 min.^21^ The themes and questions outlined in the topic guide were used flexibly and adapted as interviews took place and new information was revealed by participants. In instances where participants were not proficient in English, the questions were translated into their preferred language (French or Portuguese) during the interview. For French interviews, a translator was used, with the video conferencing platform recording and transcribing the interview. The transcription was cross-checked against the recording by a native speaker. For Portuguese interviews, the verbal responses were automatically transcribed by the video conferencing platform and the recording and transcription were cross-checked. The transcription was then transcribed back into English and checked by a native speaker.

### Data analysis

Transcripts for both studies were uploaded to and analysed in NVivo (Lumivero, Denver, CO, USA). Data analysis was conducted concurrently with the interview process, so that emerging themes could inform the questions asked in forthcoming interviews.^21^ A thematic analysis was performed to identify common salient experiences of NTD stakeholders, guided by Braun and Clarke’s six steps.^22^ Codes and themes were identified both a priori using a deductive approach based on the research question, objectives and themes developed for the interview topic guide and a posteriori using an inductive approach to recognise any unplanned or emergent themes that emerged from interviews.^23^ After completion of the interviews, the transcripts were independently reviewed and analysed by a second researcher to finalise the theme and subtheme analysis.

## Results

For study 1, 7 of 11 invited key stakeholders working on NTD programs in Nigeria participated (63.6% response rate). They were mostly males and worked at the Ministry of Health or for a NGO, with a focus on trachoma (Table 1). For study 2, 7 of 17 invited NTD program managers working for the Ministry of Health, each in a different country and working on a range of NTDs, participated (41.2% response rate), including four males and three females, with 6–17 y of experience. Specific countries are referred to as countries 1–7 (Table 1). Study 2 countries have not been specified in order to maintain participant anonymity, in line with the study ethics approval.

**Table 1.** Summary of participants’ gender, organisation type and NTD focus.


Participant	Gender	Organisation type	NTD(s) focused on in role
Study 1. Nigeria			
1	Male	Ministry of Health	Trachoma
2	Male	Ministry of Health	Lymphatic filariasis, onchocerciasis, trachoma, schistosomiasis, soil-transmitted helminthiasis
3	Female	NGO	Trachoma
4	Male	NGO	Trachoma
5	Male	NGO	Trachoma
6	Male	NGO	Trachoma
7	Male	NGO	Trachoma
Study 2. Multiple countries			
1	Male	Ministry of Health	All NTDs
2	Male	Ministry of Health	Lymphatic filariasis, onchocerciasis
3	Female	Ministry of Health	Trachoma
4	Female	Ministry of Health	Trachoma, lymphatic filariasis, schistosomiasis, soil-transmitted helminthiasis, scabies, snakebite
5	Male	Ministry of Health	Trachoma, Guinea worm, mycetoma
6	Female	Ministry of Health	Trachoma, lymphatic filariasis
7	Male	Ministry of Health	Trachoma

### Thematic analysis

**Figure 1.**
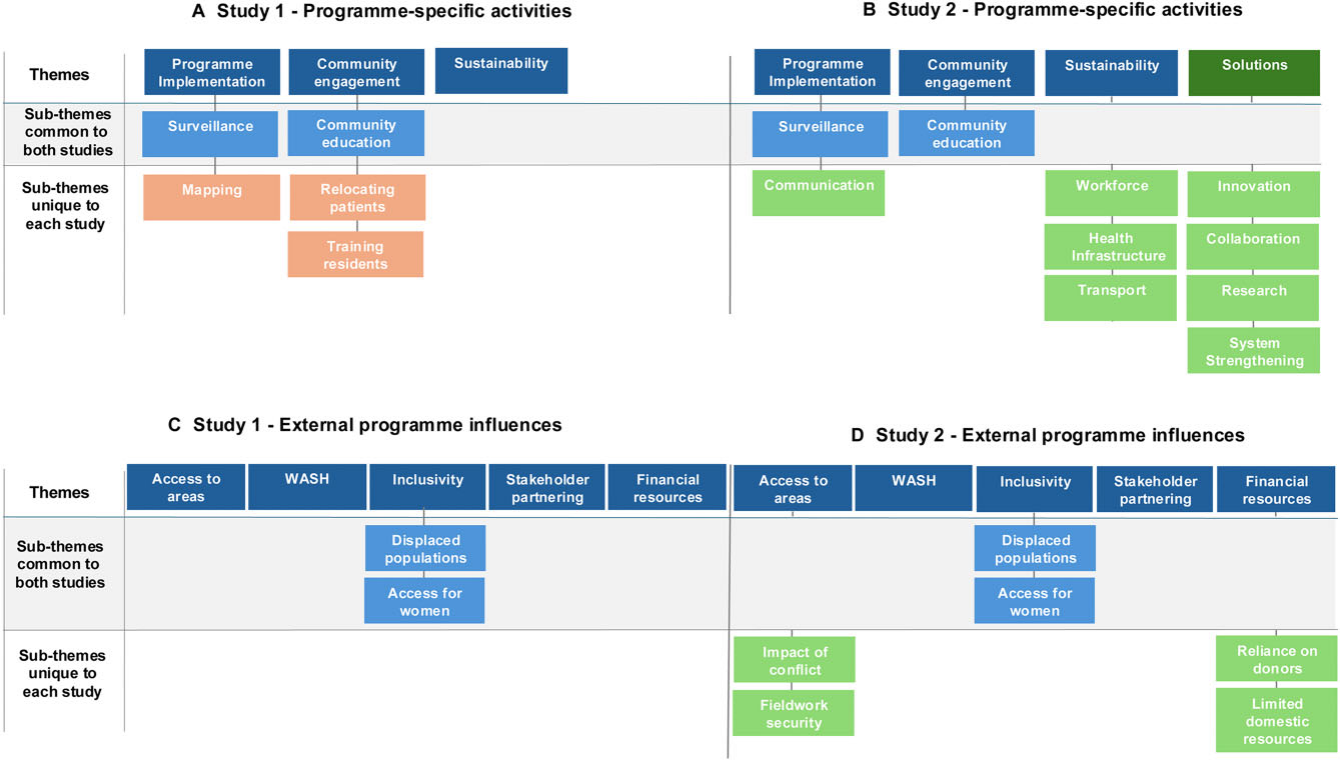
Main and subthemes in both qualitative studies. Dark blue: common themes between both studies; light blue: common subthemes between studies; dark green: unique theme identified from study 2; light orange: unique subthemes identified from study 1; light green: unique subthemes identified from study 2.

Two key categories emerged from the thematic analysis: program-specific challenges and external program influences (Figure 1). Within each category, several themes and subthemes emerged from the interviews; some were common across both studies, whereas some were unique to either study 1 or study 2.

### Program-specific challenges category

There were three common program-specific challenge themes between both studies: program implementation, community engagement and sustainability. Study 2 also identified potential solutions as a main theme, with four subthemes related to innovation, collaboration, research and health system strengthening. Table 2 summarises the program-specific challenges reported by each participant across the two studies. In addition, there were nine subthemes: two were common between the studies (surveillance and community education); study 1 reported mapping, relocating patients and training residents; and study 2 reported communication, workforce, health infrastructure and transport (Figure 1). Selected key quotes are in Boxes 1–4.

**Table 2.** Summary of the program-specific challenges and external program influences reported by participants across the two studies.

	Program-specific challenges			External program influences				
Participant	Program implementation	Community engagement	Sustainability	Access to insecure areas	Stakeholder partnering	Inclusivity	WASH	Funding
Study 1. Nigeria								
1	X	X		X	X	X	X	
2	X	X	X	X	X	X	X	X
3		X	X		X		X	
4	X	X	X		X	X	X	
5		X		X	X	X	X	
6	X	X			X	X	X	X
7	X	X	X		X	X	X	
Study 2. Multiple countries								
1	X	X	X	X	X	X	X	X
2	X	X	X	X			X	X
3	X	X	X	X				
4	X	X		X	X	X	X	X
5		X		X		X	X	X
6		X	X	X			X	X
7	X	X		X	X	X		

### Program implementation (common theme across the two studies)

Ten participants highlighted program implementation as a challenge in areas of insecurity. Selected key quotes are in Box 1.

#### Surveillance (common subtheme)

Six participants revealed the barriers that insecurity presents to achieving effective surveillance. While participants noted that there is a great need for surveillance surveys to determine whether and what type of interventions are needed in each district, such data can be difficult to obtain in insecure areas as it is dangerous for health personnel to go to these areas.

#### Mapping (study 1 subtheme)

Four participants discussed the importance of partnering with key stakeholders through baseline surveys (mapping) to optimise NTD interventions in insecure regions. Significant collaborations with the ministries of environment, education and water resources have benefited NTD programs by supporting activities of the National Trachoma Task Force.

#### Communication (study 2 subtheme/linked to health infrastructure subtheme)

Six participants highlighted how telephone lines and internet services in conflict-affected areas may be disrupted for significant periods of time, rendering the coordination of activities and information sharing between different program areas nearly impossible and hindering implementation. This is linked to the study 2 health infrastructure challenges subtheme.

Box 1. **Selected quotes on program implementation challenges.**

**Surveillance**

‘Surveillance surveys to measure the effect or your results of your intervention…So in areas in districts that have security challenges, it’s very difficult to undertake these surveys’. (Study 1, Nigeria, trachoma)

‘It’s difficult for you to measure appropriately or accurately, what are the results of your interventions. So, we have issues in terms of getting data for service in security challenged areas’. (Study 1, Nigeria, trachoma)

‘Despite the challenges, the programme has seen successes through effective strategies like using mobile devices for surveillance and hit-and-run methods for surgeries’. (Study 1, Nigeria, trachoma)

**Mapping**

‘So that’s why mapping is key. How do we do the mapping? As I said, we try to identify the key stakeholders what can we add, that can help us because of the issue of security’. (Study 1, Nigeria, lymphatic filariasis, onchocerciasis, trachoma, schistosomiasis, soil-transmitted helminthiasis)

**Communication (infrastructure)**

‘Sometimes internet is disconnected for a year for completely affected areas, but if the economy allows us then we use satellite communication. Even phone communication is impossible in conflict affected areas and, 100% we can’t access that area, unless there is satellite communication’. (Study 2, Country 1, all NTDs)

‘Power stations and telecommunications were vandalised, which temporarily isolated the region and disrupted communication’. (Study 1, Country 4, trachoma, lymphatic filariasis, soil-transmitted disease, snakebite, schistosomiasis, scabies)

### Community engagement (common theme across the two studies)

All 14 participants highlighted the need for community engagement in areas of insecurity to implement successful NTD programs. Selected key quotes are in Box 2.

#### Community education (common subtheme)

Five participants raised the importance of promoting community education in areas of conflict to disseminate information and improve awareness about the diseases and treatments alongside NTD programs; for example, educating the community about the benefits and potential side effects of the drugs and maintaining high training standards for drug distributors to ensure there are limited side effects or reactions.

#### Relocating patients (study 1 subtheme/potential solution)

Four participants highlighted the need to relocate patients to secure locations for effective trachoma surgeries. This process involves organising transport, conducting surgery in a safe area and providing aftercare advice. In Nigeria, the program funds the transport of patients from high-risk areas for surgery and post-surgery treatments.

#### Training residents (study 1 subtheme)

Four participants highlighted the benefit of recruiting and training local residents as community health workers to distribute drugs and collect data (using digital technologies in some cases) in areas of insecurity. Such individuals are likely to have greater access to populations in highly insecure areas.

Box 2. **Selected quotes on community engagement challenges.**

**Community education**

‘Another obstacle is the lack of awareness about certain diseases, with communities and political actors often unaware of neglected diseases, attributing health issues to cultural factors. Overcoming this challenge involves leveraging community knowledge and traditional networks, using local languages to disseminate information and educate populations’. (Study 2, Country 6, trachoma, lymphatic filariasis)

‘They feel that if they are with you, maybe the bandits or the whatever they may even be part of the targets’. (Study 1, Nigeria, trachoma)

‘We have to mobilise them clear clearly on the programme, let them understand the benefit they are going to have as a result of the medication we’re going to provide to them’. (Study 1, Nigeria, trachoma)

**Relocating patients (potential solutions)**

‘Sometimes if the security is intense, we ask those people to come to another place. We relocate them to another place based on discussion with them’. (Study 1, Nigeria, lymphatic filariasis, onchocerciasis, trachoma, schistosomiasis soil-transmitted helminthiasis.)

**Training residents (potential solutions)**

‘We train all our staff in different security management levels, from basic security to hostile environment and defensive driving’. (Study 1, Nigeria, trachoma)

‘Train just the people who are residing in those areas because they understand the security situation very well. They are known to the locals, so they can easily get your data’. (Study 1, Nigeria, trachoma)

‘Residents of insecure areas are also being trained to collect data and provide updated security information to the NTD programme officers. Training involves using electronic data collection systems such as CommCare and KoboCollect’. (Study 1, Nigeria, trachoma)

### Sustainability (common theme across the two studies)

Eight participants highlighted the importance of promoting sustainable NTD programs, particularly in areas of insecurity, but provide limited further information, particularly for study 1, where no subtheme could be identified. Selected key quotes are in Box 3.

#### Workforce (study 2 subtheme)

Two participants shared their experiences of the significant difficulties that arise when the programs are understaffed due to migration caused by conflict and how this poses a threat to the long-term sustainability of their program.

#### Health infrastructure (study 2 subtheme)

Three participants highlighted the impact of damage to health infrastructure in insecure regions, significantly impacting NTD control initiatives. Damage and inaccessibility to health facilities was reported to severely limit their ability to provide healthcare services to impacted communities.

#### Transport (study 2 subtheme)

Three participants reported that transport infrastructure, such as roads and bridges, is often damaged and rendered impassable due to conflict-related incidents and may hinder medical supply delivery and program staff movement. This creates difficulties in accessing remote locations where NTDs are widespread.

Box 3. **Selected quotes on sustainability challenges.**

‘Sustainability is now the issue. Because when these people are not there, I don’t know how some of the programmes continue, even though we have made a lot of progress in terms of elimination’. (Study 1, Nigeria, trachoma)

**Workforce**

‘Regrettably, there have been disruptions due to personnel shortages and the consequential migration of staff’. (Study 2, Country 3, trachoma)

**Infrastructure**

‘So, we are really suffering even if the war is stopped and it is safe to travel in those areas, the health facilities are damaged, looted, medicines are looted as they have been in the war for many years. Even after the war has stopped it’s not an easy task to resume the service because everything is damaged and distracted and one has to start from the beginning’. (Study 2, Country 1, all NTDs)

**Transport**

‘Challenges faced during a peak time in areas heavily affected, which were inaccessible due to war’. (Study 2, Country 4, trachoma, lymphatic filariasis, soil-transmitted disease, snakebite, schistosomiasis, scabies)

### Potential solutions (study 2–only theme)

The potential solutions for the challenges affecting NTD programs were not widely reported but were broadly raised in relation to innovation, collaboration, research and health system strengthening. Selected key quotes are in Box 4.

#### Innovation (subtheme)

Five participants reported that innovation is crucial in combating NTDs in conflict-affected areas. They emphasised the importance of integrating advanced technologies like biometric fingerprint recognition. Additionally, promoting sanitation marketing, such as local detergent manufacturing and toilet construction, was reported.

#### Collaboration (subtheme)

Five participants reported that collaboration with non-NTD stakeholders, particularly humanitarian agencies, NGOs (e.g. UNICEF), security operators and the military, was an important solution, as it helped in planning and executing program activities and gaining access to insecure areas while also ensuring safety.

#### Research (subtheme)

Two participants mentioned that conducting research could be crucial in addressing the numerous obstacles encountered by NTD programs in conflict settings. They emphasised that research is paramount, as it not only enhances understanding of the complex nature of various scenarios but also provides invaluable guidance in devising effective strategies to mitigate the impact of conflicts on different aspects of society.

#### Health system strengthening (subtheme)

Four participants highlighted the need for health system strengthening for effectiveness and success of healthcare initiatives, particularly in areas susceptible to conflicts. They emphasised pre-emptive planning, collaborating with the education department and tackling fundamental necessities such as WASH to significantly strengthen systems. In turn, enhancing overall communal health, having flexibility in timelines and being ready to adjust to evolving conditions were considered vital.

Box 4. **Selected quotes on potential solutions.**

**Health system strengthening**

‘The first thing is the system strengthening, we must strengthen the system so that we will be in a good position to cope up when conflicts happen, we could continue work in the absence of the external support’. (Study 2, Country 2, lymphatic filariasis and onchocerciasis)

**Collaborations**

‘…means including military personnel to facilitate their work. Collaborating with humanitarian organizations …to gain a better understanding of the actual conditions in these areas…This information is crucial for planning effective interventions. This collaboration with the military as an innovation, recognizing that waiting for change without taking proactive steps is not a viable option’. (Study 2, Country 4, trachoma, lymphatic filariasis, schistosomiasis, soil-transmitted helminthiasis, scabies, snakebite)

‘We closely collaborate with security operators to ensure thorough planning and execution’. (Study 2, Country 5, trachoma, Guinea worm, mycetoma)

‘One challenge involved transportation for field teams [in conflict affect areas], which was partially resolved with support from a non-governmental organization providing drivers and some financial and material resources.’ (Study 2, Country 6, trachoma, lymphatic filariasis)

‘…when dealing with security challenges in certain areas, we convene special planning meetings, involving traditional rulers, district representatives, LGAs, and experts from the water and sanitation sector…We seek their insights and opinions, particularly regarding safe places to conduct our service interventions. If any areas are deemed unsafe, we take their advice seriously and adjust our plans accordingly. These planning meetings are invaluable as they complement our security checks and often provide crucial information that helps us navigate complex security situations effectively…It’s crucial for everyone to collaborate and contribute to ensuring safety during program implementation’. (Study 2, Country 7, trachoma)

### External program influences

There were five main external program influence themes, which were all common to both studies: access to insecure areas, WASH, inclusivity, stakeholder partnering and financial resources (Figure 1C, D and Table 2). There were six subthemes; two were common between the studies (displaced populations and access to women), whereas study 2 also raised the themes of impact of conflict, security, reliance on donors and limited domestic resources (Figure 1). Selected key quotes are in Boxes 5–8.

### Access to insecure areas (common theme across the two studies)

Ten participants expressed the barriers encountered by their NTD programs when trying to gain access to areas of insecurity while also maintaining the safety of their healthcare workers and program officers. Participants highlighted the importance of collaborating with security personnel to continually assess the security situation in their program areas. Selected quotes are presented in Box 5. These were also highlighted as potential solutions in study 2 (Box 4).

#### Impact of conflict (study 2 subtheme)

Six participants revealed the impact of conflict on NTD program execution. They highlighted the difficulty of reaching conflict-affected areas due to unstable conditions, blockades and personal security risks, which not only hamper activities but endanger their teams. Displacement of large populations during conflicts exacerbates the spread of illnesses and obstructs disease management efforts. Additionally, achieving primary access within NTD programs is complicated by cross-border issues and regional instability, leading to missed treatment cycles for affected populations.

#### Fieldwork security (study 2 subtheme)

Three participants discussed the complications of involving security personnel for program implementation in areas of conflict and relying on security forces for clearance to access various regions, adding complexity to their tasks. They highlighted that collaborating with military forces in unstable regions is challenging, requiring expertise and often generating apprehension among the local community.

Box 5. **Selected quotes on access to insecure areas.**

‘We work in the most complex and security-challenged LGAs [Local Government Areas], requiring military approval for deploying resources’. (Study 1, Nigeria, trachoma)

‘So, before we do any activity in any security challenge area, we usually do a security assessment, to see if those areas, those districts are actually safe’. (Study 1, Nigeria, trachoma)

‘The teams now required the inclusion of a security personnel. Security considerations dictated our daily plans’. (Study 2, Country 3, trachoma)

**Impact of conflict**

‘Even currently there is an ongoing insecurity in different parts of the country. So, we have NTD care areas which are not currently accessible by the national program’. (Study 2, Country 1, all NTDs)

‘Our primary challenges primarily revolve around cross-border issues, ongoing conflicts, and insecurities in the regions where we conduct our programs’. (Study 2, Country 5, schistosomiasis, soil-transmitted helminthiasis, onchocerciasis, lymphatic filariasis, trachoma)

**Fieldwork security**

‘It led to increased costs due to the expanded team size. Previously consisting of only two individuals, the teams now required the inclusion of a security guard’. (Study 2, Country 4, trachoma, lymphatic filariasis, soil-transmitted disease, snakebites, schistosomiasis, scabies)

‘So, before we do any activity in any security challenged area, we usually do a security assessment, to see if those areas, those districts, are actually safe’. (Study 1, Nigeria, trachoma)

### WASH (common theme across the two studies)

Twelve participants discussed the importance of sustaining and promoting WASH activities to support the outcomes of NTD programs in conflict-affected areas, including working with WASH stakeholders at the coordination level to strengthen capacity for resources. This included the promotion of WASH-related messages to encourage hygiene practices and reduce rates of open defecation. Selected quotes are presented in Box 6.

### Inclusivity (common theme across the two studies)

Ten participants identified the importance of inclusivity for marginalised groups, such as internally displaced persons, refugees and women, in the planning and implementation of their NTD programs, particularly in areas of insecurity. Two participants highlighted the importance of leaving no one behind as they implement their initiatives. Selected quotes are presented in Box 6.

#### Displaced populations (common subtheme)

Ten participants noted the difficulties in identifying internally displaced populations and implementing successful NTD programs in areas with these populations, which are often areas of high poverty with unsanitary conditions.

#### Access for women (common subtheme)

Nine participants discussed the difficulties women face in accessing NTD treatment programs. Three participants noted the benefit of recruiting female healthcare workers and surgeons to mitigate this. It was also noted that women face barriers accessing clean water, further exacerbating the spread of NTDs. Such issues were prevalent in areas of insecurity.

Box 6. **Selected quotes on WASH and inclusivity.**

**WASH**

‘For us, the collaboration with security agencies, environmental ministry, and water resources ministry, we’ve been working well with them’. (Study 1, Nigeria, trachoma)

‘…migration poses a significant challenge. Moreover, we grapple with WASH, with low water and sanitation coverage’. (Study 2, Country 5, trachoma, Guinea worm, mycetoma)

**Inclusivity**

‘We do operate refugee camps in…, hosting individuals from neighbouring war-torn countries such as…, …, … and…These refugee camps are distinct entities when it comes to our neglected tropical disease (NTD) programs’. (Study 2, Country 5, trachoma, Guinea worm, mycetoma)

‘Pinpoint the difficulties encountered, particularly in isolated communities where women may find it hard to obtain clean water, which can exacerbate the spread of neglected tropical diseases’. (Study 2, Country 4, trachoma, lymphatic filariasis, soil-transmitted disease, snakebite, schistosomiasis, scabies)

‘In certain regions, we are strategically planning and collaborating with security forces to ensure that we can access hard-to-reach areas affected by conflicts, leaving no one behind as we implement our initiatives’. (Study 2, Country 5, trachoma, Guinea worm, mycetoma)

**Displaced populations**

‘The goal is to help those who have fled from distressing situations, and it would be beneficial to understand the scale of the population affected by the conflict to plan interventions effectively. However, the issue is complicated by the fact that these displaced people do not remain stationary, which adds another layer of complexity to the situation’. (Study 1, Country 4, trachoma, lymphatic filariasis, soil-transmitted disease, snakebite, schistosomiasis, scabies)

### Stakeholder partnering (common theme across the two studies)

Ten participants highlighted the significance of mapping and partnering with key stakeholders working in and around areas of insecurity to remove barriers to implementing successful NTD programs. Key partnerships, including with the Ministry of Environment, Ministry of Education and the Ministry of Water Resources, have greatly benefitted NTD programs in areas of insecurity. Selected quotes are presented in Box 7.

Box 7. **Selected quotes on stakeholder partnering.**

‘We’ve been able to map some areas because we have to work with an International Organisation of Migration, and other such agents, UN agencies that help in you know, help to track people that are displaced’. (Study 1, Nigeria, trachoma)

‘We had cooperation with the security in the state, we had cooperation with the stakeholders, the state Ministries of Health, and various community leaders’. (Study 1, Nigeria, trachoma)

### Financial resources (common theme across the two studies)

Seven participants highlighted the financial limitations NTD programs face. This included the issue of organisation dependence on external funding because of limited domestic resources, particularly in countries experiencing ongoing conflicts or economic challenges and subsequent donors not investing their funding in the most effective areas of NTD programs. It was revealed that donors often fund MDA programs but provide little funding for surveillance and impact assessment for programs in conflict-affected areas, which would have the greatest benefit. Selected quotes are presented in Box 8.

#### Reliance on donors (study 2 subtheme)

Four participants highlighted how NTD control programs seek support from external funding sources like international organisations, NGOs and bilateral donors. These entities provide financial backing for NTD control measures if a strategic plan and stability are in place. The interviewees highlighted their reliance on medication donations from pharmaceutical companies, especially for MDA. This dependence on external funding is often a result of limited domestic resources, particularly in countries experiencing ongoing conflicts or economic challenges.

#### Limited domestic resources (study 2 subtheme)

Two participants discussed that regions are often impacted by fluctuating economies, hindering governments from dedicating adequate resources to public health endeavours such as NTD control initiatives. These conflict areas often face competing financial priorities, which can lead to inadequate funding for healthcare, an issue that becomes especially pronounced in conflict-affected regions.

Box 8. **Selected quotes on financial resources.**

‘Sustainability is now the issue. Because when these people are not there, I don’t know how some of the programme continues, even though we have made a lot of progress in terms of elimination.’ (Study 1, Nigeria, lymphatic filariasis, onchocerciasis, trachoma, schistosomiasis, soil-transmitted helminthiasis)

‘Our activities are also influenced by donor conditions, as some donors require stability before providing funds’. (Study 2, Country 4, trachoma, lymphatic filariasis, soil-transmitted disease, snakebite, schistosomiasis, scabies)

‘It led to increased costs due to the expanded team size. Previously consisting of only two individuals, the teams now required the inclusion of a security guard’. (Study 2, Country 4, trachoma, lymphatic filariasis, soil-transmitted disease, snakebite, schistosomiasis, scabies)

## Discussion

This article sheds light on the substantial hurdles that NTD programs face in conflict-torn areas of sub-Saharan Africa. The interviews with key informants were an effective qualitative approach for obtaining direct stakeholder information and their experiences on the additional pressures related to NTD control and elimination. We found a combination of common and unique challenges, which may be practically addressed through research and the development of a set of standardised strategies and subsets of bespoke strategies to help meet the needs of individual countries. Common challenges included program implementation, community education, sustainability, surveillance and community engagement. There are also some study-specific challenges. Study 1, conducted in Nigeria with a primary focus on trachoma, identified three challenges, whereas Study 2, conducted across multiple countries and NTD programs, reported a wider range of unique challenges. This highlights the importance of considering the national program context in conjunction with other interconnected complex challenges and humanitarian emergencies.^24^

The primary challenges specific to NTD programs were found to be operational and logistical, related to activities such as surveillance, mapping, community education and communication. The limited access to affected populations, damage to healthcare infrastructure and poor communication technology impacted the delivery of essential services, as found with other diseases in other studies.^7,25,26^ These were potentially exacerbated by population displacement, staff migration and shortages due to security risks. Selected programs adapted to these circumstances, for example, by moving patients or training residents and community health workers. The extent of the impact on health workers, their work and highly vulnerable populations, such as refugees, is largely unknown and needs further research. The development of an NTD-specific framework, including risk assessments and mitigation action tools, is needed to help identify pathways of threats and methods of adaptation, community engagement and program and health worker optimisation. Lessons may be learned from other programs.^27–30^

Security risks are the most pressing external challenge, with unpredictable violence and blockades restricting access to endemic areas, which increases the risk of programs being unable to ‘leave no one behind’ and meet global NTD goals.^13,30–32^ Conflict-related displacement complicates the situation, as populations are unstable and often move to areas that lack basic resources and services, such as WASH. This can increase the risk of transmission of infectious diseases such as cholera, which may divert human and financial resources.^6,9,33,34^

Recent evidence in Nigeria has examined ways to improve inclusiveness and access for displaced populations, highlighting success through collaboration with a State Emergency Management Agency to map camps and the training of displaced persons to improve access to treatment.^30^

The dependency on external funding leaves NTD and other global health programs vulnerable to shifts in international financial support, exemplified in dramatic reductions in US funding to WHO.^35^ These external pressures necessitate urgent attention to health financing, resource mobilization and cross-sectoral collaboration, which align with the three fundamental pillars of the NTD Road Map 2021–2030: accelerating programmatic action, intensifying cross-cutting approaches and changing operating models and culture to facilitate country ownership.^2^ Being aware of how donor priorities may change is important, and forging new collaborations with the WASH and humanitarian sectors to maximise limited resources warrants further consideration.^24^ The critical linkage between NTDs and WASH has already been recognised, with a global strategy, guidelines and other key updates available.^36^ These need to be promoted in conflict areas.

Addressing the challenges in conflict zones requires a combination of strategic interventions. Conflict resolution efforts, such as peace-building initiatives, are essential to ensure the safety of healthcare staff and accessibility to affected regions.^37^ These are beyond the scope of national NTD programs; however, their efforts may be combined with non-state officials and implementing partners to strengthen health worker preparedness and healthcare infrastructure, including logistics management and communication systems. In addition, collaboration with the education sector and adoption of new technologies will support the healthcare workforce.^24,38,39^ There are a wide variety of resources available on the WHO learning resource hub (https://openwho.org/) regarding emergencies as a starting point for crisis management.

Moreover, fostering stronger partnerships with stakeholders, including United Nations agencies and NGOs from a variety of sectors, is vital to creating sustainable solutions for NTD control.^24^ It was reported in this study that NTD programs in Nigeria partnered with security operators to conduct security assessments to determine when and how these areas can be entered. Collaboration with security personnel has been shown to be effective for other health programs, such as in Borno State of Nigeria, where it increased access to insecure areas and improved evaluation indicators for polio programs.^26^ As such, participants in this study highlighted how vital it is to partner with security personnel working in areas of insecurity to enable the safest and most effective interventions.

The main limitations of this study included its potential biases arising from language barriers, relatively small sample size and the focus on countries with high conflict levels, which may limit the generalisability of the findings. Furthermore, most participants were from health ministries and only linked to a few NTDs (with trachoma, in particular, overrepresented), which limited potential diversity of perspectives. Notwithstanding these limitations, use of purposive sampling and digital online interviews offered a flexible, cost-effective foundation for further studies to be conducted in similar contexts across many countries. Further, the study will help develop policies and guidelines related to mapping, surveillance and response strategies in conflict-affected areas^11,12^ and identify key research priority themes, such as those summarised in Table 3, related to resilience, knowledge and capacity, vulnerable populations, new technologies and community.

**Table 3.** Summary of key research priority areas in conflict-affected areas.

Priority area	Description
NTD-specific framework	Develop a practical framework by determining what strategies are needed, who needs to be involved and how they may be implemented to enable programs to better mitigate, prepare, respond and report
Knowledge transfer and capacity strengthening	Develop effective materials and methods for educating and disseminating best practices and building local expertise and capacity that are bespoke to unique challenges
Vulnerable populations and equity	Research on the healthcare and program needs of vulnerable populations, including displaced and marginalised communities, to address inequalities
New technologies and innovation	Assess the potential and scale-up of new technologies, such as digital tools, to support diagnosis, treatment, reporting, management and communication
Community engagement	Evaluate the effectiveness of community-centred solutions and local health representation and participation for improving NTD program activities

## Conclusions

NTD programs in conflict-affected sub-Saharan African countries face many interconnected program-specific and external challenges. Key solutions include cross-sectoral partnerships, community engagement, health system strengthening, innovation and research. There is an urgent need to develop practical strategies to support programs so that progress can be made towards the NTD Road Map 2021–2030 control and elimination goals.

## Data Availability

Data are available upon reasonable request to the corresponding author.
